# Patients’ Use and Perceptions of a Drug-Drug Interaction Database: A Survey of Janusmed Interactions

**DOI:** 10.3390/pharmacy9010023

**Published:** 2021-01-19

**Authors:** Hanna Justad, Ylva Askfors, Tero Shemeikka, Marine L. Andersson, Tora Hammar

**Affiliations:** 1 Hälso- och sjukvårdsförvaltningen, Region Stockholm, 112 18 Stockholm, Sweden; ylva.askfors@sll.se (Y.A.); tero.shemeikka@sll.se (T.S.); 2 Department of Laboratory Medicine, Division of Clinical Pharmacology, Karolinska Institutet, Karolinska University Hospital, 141 57 Huddinge, Sweden; marine.andersson@sll.se; 3 Department of Medicine and Optometry, The eHealth Institute, Linnaeus University, 391 82 Kalmar, Sweden; tora.hammar@lnu.se

**Keywords:** drug-drug interaction database, DDI, public, medication information, interaction checker, information needs

## Abstract

Janusmed interactions is a drug-drug interactions (DDI) database available online for healthcare professionals (HCP) at all levels of the healthcare system including pharmacies. The database is aimed at HCP but is also open to the public for free, for those individuals who register for a personal account. The aim of this study was to investigate why and how patients use the database Janusmed interactions, how they perceive content and usability, and how they would react if they found an interaction. A web-based questionnaire was sent by email to all users who had registered for Janusmed interactions as a “patient” (n = 3219). A total of 406 patients completed the survey (response rate 12.6%). The study shows that there is an interest among patients to use a DDI database to check their own or a relative’s medication. The respondents found the database easy to use and perceive they understand the information aimed at HCP. Most patients stated they would talk to their HCP if they found an interaction and not adjust their treatment by themselves. However, the respondents in this study are actively searching for information and seem to have high health literacy. Thus, the findings are not generalizable for the general population.

## 1. Introduction

Drug treatment is an essential part of healthcare, to cure illness and to maintain physiological functions. There are advanced therapies available to treat severe conditions, many patients are on life-long medication and there is a growing group of patients using many drugs for a long time. An important feature to take into account is drug-drug interactions (DDI) that may cause adverse drug reactions (ADR) and therapeutic failure, both of which in turn may cause human suffering and extended treatment periods, which is expensive at a societal level. [1,2,3,4] Many DDI are preventable, either by the choice of drug or by dosage modification. Drug treatment should therefore be guided by evidence-based recommendations on how to handle DDI [3,5,6,7,8,9].

**Figure 1:**
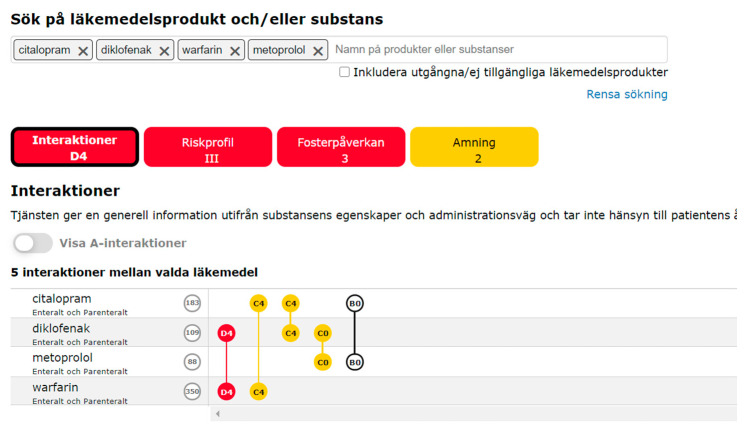
User interface Janusmed interactions (version 3.0.11) (Region Stockholm, Stockholm, Sweden)

Janusmed is a collection of databases for safe drug treatment [10]. Janusmed interactions (formerly known as Sfinx) is a DDI database aimed at healthcare professionals (HCP), which has been available in Sweden since 2007 [3]. The database is used to check for pharmacokinetic DDI and provides information on potential consequences and a recommendation for HCP on how to handle the interaction. Janusmed interaction may be embedded in electronic health records (EHR) as one component in clinical decision support systems (CDSS) but is also available online to everyone who registers as a user [3]. The Janusmed interaction database contains information about DDI between active substances in drug products available on the Swedish market. Although DDI (including herbal remedies) is the core of the database, some drug-alcohol and drug-food interactions are also presented. The database covers clinically relevant pharmacokinetic interactions. The interactions are classified as A–D, showing the interactions’ relevance together with a number 0–4 indicating the interactions’ grade of documentation, see example in Figure 1. Some pharmacodynamic interactions are included because of high clinical relevance, but pharmacodynamic interactions are mainly covered in the knowledge database Janusmed riskprofile (formerly known as PHARAO), which is complementary to Janusmed interactions but available to HCP exclusively [11]. The Janusmed collection also includes knowledge databases on drugs and lactation, teratogen effects, renal function, sex and gender [12,13,14]. In the online version of Janusmed interactions, the user logs in and enters two or more drugs in order to then receive a graphic presentation of potential DDI, if there are any [10].

The database is an available source of information on DDI for all healthcare providers and pharmacies across the country, which is a conscious choice in a shared effort to provide patients with consistent information and promote adherence to treatment [15]. CDSS may improve practitioner performance; however, for such systems to work the data must be of high quality and presented in a user-friendly way to assure both safety and the accuracy of the system [3,16,17]. Several studies have been published assessing professional DDI-checkers, although the existence of and knowledge about DDI-checkers for patients/public is sparse [7,8,18,19,20].

There are, to our knowledge, no interaction databases aimed at patients/public available in Sweden. Drug information is available in FASS (Pharmaceutical Specialities in Sweden), an information database of drug facts on medicines registered to be used and purchased in Sweden. The information in FASS is provided by pharmaceutical companies and is presented in two editions, one aimed at the public and one aimed at healthcare professionals and veterinarians. The edition aiming at the public contains patient information leaflets with some information regarding DDI [21]. 

The internet is a common source for health information, and drug information is among the most commonly searched health information topics [22]. Health information seeking is important because patients take an active role in healthcare, and actively seeking information about health-related issues can be a key coping strategy in health-promotive activities and psychosocial adjustment to illness or condition [23,24]. Previous research suggests that patients complement information from healthcare professionals with information they find on the internet. The demand for health information also challenges society to provide reliable and easily accessible sources. Incorrect information about drug treatment could have serious consequences, hence there is need for oversight to eliminate low quality and potentially harmful sources [25]. Several barriers have been identified when searching the internet for health information [26]. One such barrier is the patient’s eHealth literacy, i.e. how capable the patient is of finding, understanding and assessing information from electronic sources and applying this information to solve a health problem [27,28]. The barriers may lead to negative outcomes, such as patients not knowing which source to believe or straying from the health directives given by their HCP [29].

For patient safety and for patients to feel safe and adhere to their medication treatment, it is crucial to provide understandable information that match/fulfil the individual patient’s information needs [18]. Besides the basic information needs, i.e., which medicines to take and how and when, patients may want more information to evaluate the benefits of the prescribed drug and weigh it against their concerns. While a strong belief in benefits appears to predict adherence, a “concern belief” may lead to intentional non-adherence. It is supposed that providing subjectively desired information about medication can prevent patients’ concerns. Patients have different needs and request regarding drug information, but information about ADR and DDI are the two most commonly requested topics [18]. However, little is known about how to customize such information for patients’ needs. 

Within the elderly part of the Swedish population, many need healthcare but only 40% of people 65 years and older utilize the healthcare community services, and only 4% make digital healthcare visits. For Swedes of ages 75 years and older, the numbers are 32% and 3%, respectively. Education also seems to be of importance for accessing digital healthcare services. There is an emerging gap between high educated and low educated people, where the latter is at risk of being excluded from digital health care. An exception to this is the use of digital healthcare apps, which replace physical visits; in this case, there is no difference in education level [30,31]. 

Physicians are responsible for making sure a patient’s medication treatment is appropriate [32]. However, there are several barriers for this, such as incomplete medication lists, lack of knowledge about certain interactions and different perceptions about responsibility [3,32,33,34]. Prescribers often do not have access to all prescriptions made by other HCP due to lack of interoperability between different EHRs [35]. Patients also use OTC (over the counter drugs, available without prescription) and herbal remedies on their own, which are not on their medication list [36]. Besides the challenge of lack of overview of a patient’s medication, prescribers’ knowledge of clinically relevant DDI may vary and there is need for support systems to identify DDI and adjust inappropriate combinations [3,7,34]. Pharmacists may support prescribers in managing DDI and also play a vital role in patient education. Alongside prescribers, they are an important and trusted source of healthcare information for patients [37,38,39]. 

The aim of this study was to investigate why and how patients use the DDI database Janusmed interactions and was developed with HCP as a target group. The research questions were:

Q1: What kind of information do patients want?
Q2: How do patients perceive the content and usability?
Q3: How would patients react if they found DDI in their medication?
Q4: Is there a perceived need for a “patient version” of the database Janusmed interactions?

## 2. Materials and Methods 

### 2.1. Participants, Inclusion and Exclusion 

This survey was directed at individuals who use Janusmed interactions for private purposes. Janusmed interactions have over 48,000 registered active accounts (spring 2020). Most of them belonged to HCP (healthcare professionals), but more than 3000 accounts were registered as “Patient” or “Other”. To be able to use Janusmed interactions online, an account must be created with an e-mail address, and the registrant must state profession/role. The survey was sent to the e-mail addresses of 3219 individuals who had a registered account as “Patient” or “Other”. The survey was sent on the 17th of April and closed four weeks later on the 15th of May 2020. A reminder to answer the survey was sent after 14 days.

### 2.2. The Questionnaire

The survey was created and distributed with Questback Essentials® (Questback Sweden AB, Stockholm, Sweden). It comprised twelve questions (see Table 1), for six of which the respondent was asked to add additional information if they wanted. The questions were intended to give more information about the end-user, their perception of the usability of the database and how they would act after reading the recommendations. The last question was an NPS (Net Promoter Score) question on whether the participant would recommend Janusmed interactions to a friend or relative and was intended to measure the overall user satisfaction of the website [40].

### 2.3. Analysis 

Answers to the questionnaire were analyzed using descriptive statistics in Excel (Microsoft corporation, Washington, WA, USA) and SPSS software (IBM, New York, NY, USA). Chi-2 test was used to find any differences in answers related to age. The responses to the questionnaire in free text were analyzed and categorized using manifest content analysis methods [41]

### 2.4. Ethical Considerations

The question to participate in the study was sent to the e-mail addresses the users had registered with. In the email, they were asked to answer the questionnaire and informed that it was voluntary. When users register for an account, they are informed that the knowledge databases are aimed at HCP and that it is used by laymen at their own risk. They are also informed that the information they register is kept by the Region of Stockholm and can be used for statistics about the use of the database. However, they were not asked beforehand if they wanted to receive invitations to participate in research studies. No identifiable personal information has been collected from the respondents answering the questionnaire, and the answers cannot be linked to any individuals. The questionnaire did not include any questions about sensitive health data. Results are presented on a group level so that no individual respondents can be identified. This work was primarily planned to be part of the regular evaluation and development of the database, regarding content and usability. However, since there is limited research on this topic, we realized the value of sharing the results scientifically as well. 

## 3. Results

In total, 522 people responded to the survey. However, 106 persons were excluded after the first control question about whether they used the Janusmed interactions for work or private purposes. Another 10 respondents were excluded as they answered “never” to the question about how often they use the database. In total, 406 people completed the full survey and were included in final analysis (response rate 12.6%). All the participants were Swedish residents over 18 years old, 39.4% were between 19–39 years old, 35.8% were 40–59 years, 22.4% were 60–80 years and 2.4% were over 80 years. The respondents came from different parts of Sweden, all of Sweden’s regions were represented among the answers with a higher number of respondents from the regions with highest population and fewer from the smaller regions The majority of respondents (64.4%) reported that they used Janusmed interactions a few times a year, while 30% stated they used the database a few times a month (Table 2). There was no significant difference between age groups in how often they use Janusmed interactions.

### 3.1. Reasons for Using Janusmed Interactions

On the question of why the respondents use Janusmed interactions, the majority (83.7%) said they use it to check if their prescribed medications had any interactions (Table 3). Respondents also used it to check for interactions with OTC-drugs, food/beverages, herbal remedies and alcohol. It was rather common for respondents to use the database to help a friend or relative check for interaction or for other reasons. The younger age groups more often used Janusmed interactions to check interactions for a friend or family (*p* < 0.000), to learn about drug interactions (*p* = 0.033) and for other reasons (*p* = 0.006). 

Of the respondents, 7.4% opted to describe, in their own words, other reasons for using the database. Free text comments about reasons for using Janusmed showed that many users are students, using the database for their studies in pharmacy or medicine to learn more about DDI. There were also several respondents who answered that they are retired from a health care profession. Others described that they use Janusmed interactions to check OTC-drugs, food, herbal drugs or during pregnancy and breast feeding. 

“My doctor keeps track of my prescribed drugs. But if I buy, for example, pain killers, then I want to make sure they are ok to combine with my prescribed drugs.”

Some respondents declare they use Janusmed interaction because of a lack of trust in physicians checking DDI, so they feel they have to check that the medications they (or their relatives and friends) get are safe to combine.

“A close relative has a lot of different medicines, and I always go in and check when something is replaced” 

“My experience is that doctors have surprisingly low knowledge about interactions. More than once, I have had to point out that a proposed new drug does not go with those I already have.”

### 3.2. Patients’ Perceptions of Janusmed Interactions 

Most individuals (48.8%) who answered the survey thought Janusmed interactions contained everything they needed. The most requested areas that should be better covered were dietary supplements (35.7%), herbal drugs (27.3%) and food/beverages (24.4%). A larger proportion from the younger age group wanted more information regarding alcohol (18.2% compared with 4.0% in the age group 60 or older, *p* < 0.0001). Free text answers with suggestions on more areas from the respondents included interactions in relation to drug concentration, more unusual dietary supplements, food, tobacco and pharmacodynamic interactions. Other content that the respondents felt was missing was information about adverse effects with long term use of medications, more information about medications during pregnancy or breast feeding and more information about the interactions. 

Most respondents, 90.9%, said Janusmed interactions was easy to use, and 93.3% also thought the interaction texts were easy to understand, while only 4.2% said it was difficult. Free text answers regarding improvements of Janusmed covered content, functionality and usability. Several suggested that Janusmed interactions should be available without having to log in, that there should be user instructions, and that it should be easier to search and check interactions when you have many medications. Several also wanted to be able to save their medication list. The free comments about the information texts mainly focused on the fact that the texts are written for HCP and therefore hard to understand for laymen. More than half (58.1%) of the respondents stated they would use a version of Janusmed interactions specifically aimed at the public, if such a version existed. Although 13.1% would not use such a source, and 30% were undecided.

### 3.3. (Re) Actions When Finding an Interaction

When asked how they would react if they found a DDI, the majority (93.1%) said they would talk to the prescribing HCP. Some of the respondents (11.3%) said they would consider adjusting their treatment on their own, such as lowering the dose, while others (8.1%) would consider stopping the drug on their own. Only 4.2% would not do anything if they found an interaction. A larger proportion of the younger age group answered that they would not do anything (6.9% compared with none of the oldest age group, *p* = 0.024). Of the respondents, 7.4% stated they would do something else and further elaborated in the comments. Many said it depended on how serious the interaction was or that they would want to discuss the issue further with a pharmacist at their local pharmacy. Others would try to get in contact with their HCP but make changes in their medication if they deemed this taking too long.

“Generally I would not make changes or interrupt my medication before talking to my care center. If I found it difficult to get a hold of them, I might consider lowering the dose/pausing the treatment. It depends on the type of medicine and how serious the consequences of an interaction could be”.

Several respondents answered that their action depended on the interaction, the drugs involved or if they experience any side effects. Sometimes they adjusted their drug treatment but still contacted their HCP. 

“If two prescribed medicines interacted, I would contact my physician immediately. If my prescribed medicine interacted with OTC, I would avoid using the OTC unless I had to. In that case, I would consult my physician.”

“It depends on the interaction. If possible, I would, for example, spread out the dosing times, but for interactions that require [major] changes in the treatment, I would contact my physician.”

### 3.4. Other Information Sources Used by Patients 

Among the respondents, 53% said they did not use any other sources to check for interactions, while 47% used other sources. There was a significant difference between age groups for this question, with 56% of the youngest age group answering that they use other sources (*p* = 0.024). The most common answer in free text was that they were using FASS as an information source about interactions, both FASS for patients/public and FASS for health care professionals. Other sources included pharmacists working in pharmacies, the patient’s doctor/ prescriber, the patient information leaflet (PIL), pharmaceutical journals and lists of recommended drugs in the region the responder was located. Other internet-based sources mentioned were Evidos, Micromedex, MedicinesComplete, Medscape, FDA, EMA, and drugs.com. A few of the respondents also stated they used Google, Wikipedia, Tripsit, Flashback or “a variety of foreign sources”.

### 3.5. User Satisfaction—Net Promotor Score 

**Figure 2:**
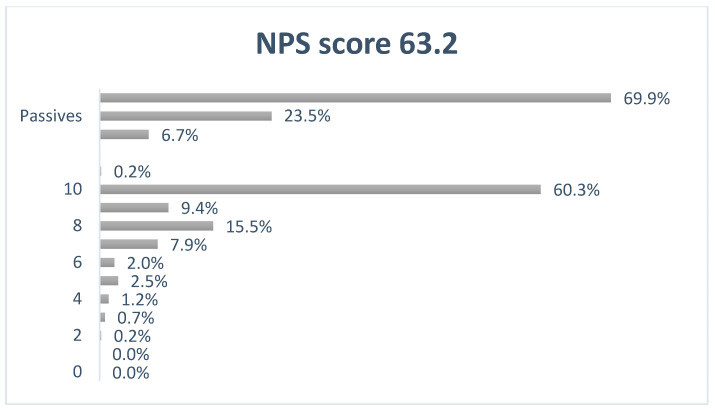
Net Promotor Score for Janusmed interactions.

Janusmed interactions received an NPS score of +63.2; 69.9% of the responders were deemed as promotors, while 6.7% were detractors and 23.5% remained passive (Figure 2). 

## 4. Discussion

This study shows that Janusmed interaction is, although aimed at HCPs, also utilized by patients wanting to check their own or someone else’s medication for interactions. These are actively searching for medical information (many used several different sources) and may therefore not be generalizable for the entire population. Most respondents found the database easy to use and said they could understand the information in the texts well enough although they were sometimes too “technical”. The majority of patients also stated they would talk to HCP if they encountered an interaction and not make changes in the treatment by themselves. The study results illustrate that there is a demand for information on DDI among laymen. 

### 4.1. Patient Information Needs

With the digital information society, health information is more readily available to patients. One group of users was retired HCP who wanted to check their own medication or help others do the same, while another was individuals mainly focused on helping friends and relatives check for interactions. This could perhaps suggest younger individuals with more computer skills helping older relatives and friends, and in the process giving them access to digital health information. In this study, 75% of respondents were younger than 60 years, which coincides with the findings that younger people use the internet more than the elderly [31]. This could be a problem since the elderly are generally prescribed more drugs. However, this study shows they might not be entirely excluded from the database thanks to younger friends and family members. One could also note that most of respondents said they used Janusmed interactions a few times a year, which might coincide with doctor appointments or collecting their prescriptions at the pharmacy. 

It has been shown earlier that patients have different information needs [18]. In this study, the majority of the respondents stated that they can understand and retrieve the information they need from the interaction text in the current form. However, they also said it would be useful to have a version aimed at laymen. There were some inquiries for more comprehensive information texts; however, few respondents gave any feedback regarding exactly how the texts could be improved. Previous work in Sweden and in other countries indicates that it is beneficial for HCP and patients to have access to information from the same database [42,43]. This can improve both communication and compliance while preventing misunderstandings. Clinical decision support systems are also more likely to be effective.

### 4.2. Sources of Information 

Many of the respondents stated they use sources other than Janusmed interactions to learn about DDI, including other digital resources. This is in line with previous research describing that patients’ sources of drug information and information about DDIs are primarily physicians, pharmacists, relatives, patient information leaflets and online sources [44,45,46]. Internationally there are different mobile apps and web pages that provide DDI services to patients, but research regarding patients use of them is limited [19,20,25]. Previous studies have shown that patients often have limited knowledge and awareness regarding DDIs [38,47]. Our finding supports the image of the respondents being active information seeking patients. It may also imply these patients will continue to try to find information if they did not have access to Janusmed interactions. This highlights the importance of making reliable sources of health information available to patients, otherwise they risk getting incorrect information about their medication, which could impact their health negatively.

### 4.3. Handling Interactions 

There is likely a difference between how patients and HCP digest and process the information in Janusmed interactions. Since HCP are educated in medicine and often have extensive work experience, they have a deeper understanding of a patient’s drug treatment and can take several other factors into consideration. They might also understand that they need complementary information and sources to get the full picture of a patient’s DDI risk, such as Janusmed riskprofile and other pharmacodynamic interactions [11]. Because of this, there are concerns about how patients would react if the database warned about an interaction. In particular, if they would make changes or stop taking their medication on their own. Our results indicate that this fear may have been unwarranted since most of the respondents stated they would talk to HCP if this happened. The respondents also seemed to distinguish between prescribed medication and OTC and assessed how important the medication was to their health. It is important to note that the risk of patients making changes in their medication is ever present and patients can stop taking or make changes in the medication for a variety of reasons such as fear of the medication or experiencing side effects. However, a DDI database aimed at the public should contain a clear message directed at the patients to consult their HCP if they do find an interaction to ensure they do not act on their own accord. 

### 4.4. Heading For a “Patient Edition” of Janusmed Interactions

The respondents in the study were comfortable handling DDI-information aimed at HCP, which indicates this patient group have high eHealth literacy. Still, 58% of them stated they would use a patient version of the database if it was available. Unfortunately, they did not provide any suggestions on how the information texts could be improved and simplified in the survey, only that they should be “simpler”. Previous studies suggest that among patients there is a need of “rather diverse but only limited information domains” regarding DDI [18]. Vingen et al [20] state that in order for patients to act as decision-makers regarding their own health, we need to empower them with information. The systems conveying this information must be usable in order for empowerment to take place. Offering a patient version of Janusmed interactions aimed at laymen would give patients access to the same DDI-information as HCP and community pharmacists. Different interaction databases may have differences in alerts [8] and recommendations, which could lead to confusion and more difficult communication if the patients are using another database than HCP. There may always be some differences in certain recommendations compared to other sources of medical information, e.g., Micromedex as mentioned above, and these differences must be valued and handled by HCP. However, both professional and public users need reliable and easily accessible information. The existence of several interfaces and applications using the same evidence-based content, such as the Janusmed database, would be a great advantage to all actors in the healthcare system [42,43]. Tailored decision support adapted for different medical specialties, pharmacists, nurses etc. in hospitals, primary care, pharmacies and patient information sources would in the end facilitate care for the patient and help compliance to treatment. It would also facilitate the regulatory processes for authorities to approve software medical devices if they are based on same evidence-based content. All in all, this could empower HCP and patients together and endorse patient safety.

### 4.5. Method Discussion

The study population presented in this paper seem to be a group of active information seeking individuals. Their answers indicate that many of them have good eHealth literacy and higher medical knowledge than average individuals. There is a trend of well-informed patients seeking out and bringing information themselves to their HCP for further discussion. These patients are often younger and/or highly educated. In the free comments a few respondents stated they studied to become HCP and might therefore have more knowledge and understanding of DDI than patients normally do. There was, however, no way to systematically exclude healthcare students from the analysis. The questionnaire had a response rate of 12.6%. We do not know how well the respondents reflect all patients using Janusmed interactions. Therefore, the study population presented in this paper may be a group of information seeking individuals with high health literacy and not generalizable for all patients in Sweden. A limitation of the study is that it is based on users’ self-reports of their experiences and not on their real-time actions when using the database. 

The strength of the study was that the survey was sent to all users with access to Janusmed interactions registered as public/laymen. The analysis indicated that for the future, it would be interesting to know more about the educational level of the responders and their internet habits in general. It would also be valuable to further explore the health literacy of the users. However, this work may be regarded a first attempt to investigate the topic. A more comprehensive study with a mixed-methods approach would provide deeper knowledge about how patients’ use and perceive Janusmed interactions.

### 4.6. Future Studies

The current study contributes to the knowledge base regarding patients taking an active role in their medication treatment. However, more research about how to provide DDI information to patients is needed. It would be valuable to further investigate user behavior based on system logs for the database to follow how it is handled by patients. Future work may investigate the effects of patients using Janusmed interactions from different perspectives. Further studies should also evaluate the effects of a patient’s version of Janusmed interaction being provided and communicated to a larger group of patients and elaborate on the design and presentations of a DDI database to these users. 

## 5. Conclusions

There is an interest among patients to use a DDI database. The respondents to this study were an information seeking group who mainly used Janusmed interactions to check their own or a friend’s or relative’s medication. They used the database primarily to check their prescribed medications but also OTC medications, herbal drugs, food and alcohol. The respondents perceived the database as easy to use with comprehensible, although sometimes a bit too technical and scientific, texts. In case they found interactions in their treatment, most respondents said they would talk to their HCP but not quit their drug treatment on their own. Respondents had some suggestions for improvements and additional features of the service, and the majority stated they would use a version of Janusmed interactions specifically aimed at patients. Future research should study how such a service can be designed to meet the needs of patients without risking their safety. 

## Figures and Tables

**Table 1:** Description of the questionnaire.

	Question	Possible Answers (Multiple Choice)	Multiple Answers Possible	Possible to Comment in Free Text
1	In which capacity do you use Janusmed interactions?	(a) Privately (b) In my profession	No	No
2	How old are you	(a) under 18 years (b) 19–39 years (c) 40–59 years (d) 60–80 years (e) over 80 years	No	No
3	In which region are you located?	(a) Blekinge (b) Dalarna (c) Gotland (d) Halland (e) Jämtland Härjedalen (f) Jönköpings län (g) Kalmar län (h) Kronoberg (i) Norrbotten (j) Skåne (k) Stockholm (l) Sörmland (m) Uppsala (n) Värmland (o) Västerbotten (p) Västmanland (q) Örebro län (r) Östergötland (s) Västra Götaland (t) located outside of Sweden	No	No
4	How often do you use Janusmed interactions?	(a) Multiple times a day (b) Once a day (c) A few times a week (d) A few times a month (e) A few times a year (f) Never	No	No
5	Why do you use Janusmed interactions?	(a) To check if my prescribed medication have interactions (b) To check if my prescribed medication interact with OTC drugs I have/plan to buy (c) To check if my OTC medication have interactions (d) To check if my medication interact with food/drink (e) To check if my medication interact with alcohol (f) To check if my medication interact with naturopathic drugs (g) To check interactions for a friend or relative (h) I look with my HCP to discuss my treatment (i) To learn more about drug interactions (j) Other	Yes	Yes
6	Are there any areas you miss/want more information about in Janusmed interaction?	(a) No, nothing (b) Food/drink (c) Alcohol (d) Herbal remedies (e) Dietary supplements (f) Other	Yes	Yes
7	Do you think Janusmed interactions is easy to use?	(a) Yes (b) No (c) Don’t know	No	Yes
8	Do you think the texts are easy to understand?	(a) Yes (b) No (c) Don’t know	No	Yes
9	If you had prescribed medication and found out they interacted how do you think you would react?	(a) Talk with my prescriber (b) Discontinue the treatment on my own (c) Make changes in the treatment on my own (d) Wouldn’t do anything (e) Other (f) I don’t know	Yes	Yes
10	Do you use any other sources to check for drug interactions?	(a) Yes (b) No	No	Yes
11	Would you use a version of Janusmed interactions specifically aimed at the public?	(a) Yes (b) No (c) I don’t know	No	No
12	On a scale from 0–10 how likely is it that you would recommend Janusmed interactions to a friend or colleague?	A scale of 0 to 10	No	No

**Table 2:** How often Janusmed interactions is used (n = 406)**.**

Q4: How Often Do You Use Janusmed Interactions?	n	%
Multiple times a day	1	0.2
Once a day	2	0.5
A few times a week	10	2.5
A few times a month	125	30.8
A few times a year	268	66.0
Total	406	100.0

**Table 3:** Reasons to use Janusmed interactions (n = 406).

Why Do You Use Janusmed Interactions?	Total (n)	Total (%)
(a) To check if my prescribed medication has interactions	340	83.7
(b) To check if my prescribed medication interacts with OTC drugs I have/plan to buy	243	59.9
(c) To check if my OTC medication have interactions	132	32.5
(d) To check if my medication interacts with food/drink	115	28.3
(e) To check if my medication interacts with alcohol	83	20.4
(f) To check if my medication interacts with herbal remedies	110	27.1
(g) To check interactions for a friend or relative	180	44.3
(h) I look with my caregiver to discuss my treatment	19	4.7
(i) To learn more about drug interactions	162	39.9
(j) Other	30	7.4

## Data Availability

The data presented in this study are available upon request from the corresponding author. The data are not publicly available due to the character of the study, please see Section 4.5.
